# Range Variability in CMR Feature Tracking Multilayer Strain across Different Stages of Heart Failure

**DOI:** 10.1038/s41598-019-52683-8

**Published:** 2019-11-11

**Authors:** Radu Tanacli, Djawid Hashemi, Tomas Lapinskas, Frank Edelmann, Rolf Gebker, Gianni Pedrizzetti, Andreas Schuster, Eike Nagel, Burkert Pieske, Hans-Dirk Düngen, Sebastian Kelle

**Affiliations:** 1 Department of Cardiology, German Heart Centre Berlin, Berlin, Germany; 2 Department of Cardiology, Charité University Medicine Berlin, Berlin, Germany; 3 German Centre for Cardiovascular Research DZHK, Partner Site Berlin, Berlin, Germany; 4 Department of Engineering and Architecture, University of Trieste, Trieste, Italy; 5 Department of Cardiology and Pulmonology and German Centre for Cardiovascular Research (DZHK) Partner Site, Göttingen, Germany; 6 Institute of Experimental and Translational Cardiac Imaging, DZHK Centre for Cardiovascular Imaging, Goethe University Hospital Frankfurt, Frankfurt am Main, Germany

**Keywords:** Cardiology, Computational biology and bioinformatics, Cardiac hypertrophy

## Abstract

Heart failure (HF) is associated with progressive ventricular remodeling and impaired contraction that affects distinctly various regions of the myocardium. Our study applied cardiac magnetic resonance (CMR) feature tracking (FT) to assess comparatively myocardial strain at 3 distinct levels: subendocardial (Endo-), mid (Myo-) and subepicardial (Epi-) myocardium across an extended spectrum of patients with HF. 59 patients with HF, divided into 3 subgroups as follows: preserved ejection fraction (HFpEF, N = 18), HF with mid-range ejection fraction (HFmrEF, N = 21), HF with reduced ejection fraction (HFrEF, N = 20) and a group of age- gender- matched volunteers (N = 17) were included. Using CMR FT we assessed systolic longitudinal and circumferential strain and strain-rate at Endo-, Myo- and Epi- levels. Strain values were the highest in the Endo- layer and progressively lower in the Myo- and Epi- layers respectively, this gradient was present in all the patients groups analyzed but decreased progressively in HFmrEF and further on in HFrEF groups. GLS decreased with the severity of the disease in all 3 layers: Normal > HFpEF > HFmrEF > HFrEF (Endo-: −23.0 ± 3.5 > −20.0 ± 3.3 > −16.4 ± 2.2 > −11.0 ± 3.2, p < 0.001, Myo-: −20.7 ± 2.4 > −17.5.0 ± 2.6 > −14.5 ± 2.1 > −9.6 ± 2.7, p < 0.001; Epi-: −15.7 ± 1.9 > −12.2 ± 2.1 > −10.6 ± 2.3 > −7.7 ± 2.3, p < 0.001). In contrast, GCS was not different between the Normal and HFpEF (Endo-: −34.5 ± 6.2 vs −33.9 ± 5.7, p = 0.51; Myo-: −21.9 ± 3.8 vs −21.3 ± 2.2, p = 0.39, Epi-: −11.4 ± 2.0 vs −10.9 ± 2.3, p = 0.54) but was, as well, markedly lower in the systolic heart failure groups: Normal > HFmrEF > HFrEF (Endo-: −34.5 ± 6.2 > −20.0 ± 4.2 > 12.3 ± 4.2, p < 0.001; Myo-: −21.9 ± 3.8 > −13.0 ± 3.4 > −8.0 ± 2.7. p < 0.001; Epi-: −11.4 ± 2.0 > −7.9 ± 2.3 > −4.5 ± 1.9. p < 0.001). CMR feature tracking multilayer strain assessment identifies large range differences between distinct myocardial regions. Our data emphasizes the importance of sub-endocardial myocardium for cardiac contraction and thus, its predilect role in imaging detection of functional impairment. CMR feature tracking offers a convenient, readily available, platform to evaluate myocardial contraction with excellent spatial resolution, rendering further details about discrete areas of the myocardium. Using this technique across distinct groups of patients with heart failure (HF), we demonstrate that subendocardial regions of the myocardium exhibit much higher strain values than mid-myocardium or subepicardial and are more sensitive to detect contractile impairment. We also show comparatively higher values of circumferential strain compared with longitudinal and a higher sensitivity to detect contractile impairment. A newly characterized group of patients, HF with mid-range ejection fraction (EF), shows similar traits of decompensation but has relatively higher strain values as patients with HF with reduced EF.

## Introduction

Heart failure represents worldwide a massive burden and, despite progresses in treatment strategies including medication and cardiac device therapy^1^, due to general aging, the absolute number of cases increased with 12% in the last decade^2^, with an anticipated prevalence increase of 46% before 2030^3^. An increased preponderance and higher mortality of patients with diastolic heart failure^3^ warrants improvements towards a more particularized therapeutic approach and follow-up.

Human heart anatomy is complex, comprising 3 distinct layers of muscular fibers with different angular orientation, starting from a longitudinal distribution toward the endocardium and progressively being interspersed by oblique fascicles with contrary helical orientation from base to the apex and, respectively, by circular fascicles towards the epicardium. This particular architecture is essential to ensure a specific pattern of motility and force generation during the cardiac cycle: descend of the mitral valve plane towards the apex, uniform decrease/increase in short-axis diameter, base to apex twisting/untwisting^4,5^. Complexity to the assessment of heart contraction increases when myocardium undergoes pathologic hypertrophic remodeling or develops microvascular dysfunction due to various systemic syndromes such as hypertension, obesity, diabetes or coronary artery disease^6^.

Being less influenced by haemodynamic forces and tethering effect of adjacent segments, strain rate imaging holds the promise to render an adequate evaluation of the spatial contractility parameters (longitudinal, circumferential and radial strain) measurable at each voxel level over the cardiac cycle^7^. Longitudinal strain is a powerful predictor of mortality in patients with acute^8^ and chronic heart failure^9^, overpassing widely used parameters such as ejection fraction (EF) or left ventricular (LV) volumes. Moreover, in patients with myocardial infarction (MI), reduced myocardial strain values are quantitatively correlated with the extent of MI and respectively, with the incidence of adverse event and prognostic^10^.

CMR feature tracking (FT) technique provides information about myocardial mechanics. Myocardial strain and strain rate are obtained from the analysis of conventional balanced steady state free precession (bSSFP) cine images^11^, with an excellent inter- observer, intra- observer agreement and high inter- study reproducibility^12,13^.

The purpose of this study was to assess the longitudinal and circumferential strain at the level of 3 distinct myocardial layers (subendocardial, myocardial and subepicardial) in HF patients and correspondent age- and gender- matched normal subjects and give an account of the variability of dimension range and discriminating diagnostic ability of these parameters.

## Methods

### Study population

The study was approved by the Ethics Committee of the Charité-University-Medicine in Berlin, complied with the Declaration of Helsinki and was registered at the German Register for Clinical Studies (DRKS) (registration number: DRKS00015615). All individuals were fully informed about the aim, rationale and detailed protocol of the study (subject information leaflet) and consequently written informed consent was obtained before participation. For this study, we included prospectively, patients with a diagnosis of HF who were subdivided according to recent guidelines^14^ into 3 groups, as follows: (1) patients with HF with preserved ejection fraction (HFpEF), where EF ≥50% but diastolic dysfunction is present (an E/e′ ≥13 and a mean e’ septal and lateral wall <9 cm/s) and plasma levels of NT-proBNP >125 pg/mL, (2) patients with HF with mid-range ejection fraction (HFmrEF), where EF = 40–49% and similar additional criteria are present, (3) patients with HF with reduced ejection fraction (HFrEF) where EF < 40%. Exclusion criteria were: more than trivial MR, uncontrolled hypertension, atrial fibrillation, incompleteness, reduced quality or presence of artifacts in the images acquired. Inclusion of patients took into account a match for age and gender distribution between the groups. For comparison a fourth, similar group of age- and gender- matched volunteers have been recruited, and underwent an identical protocol with the HF subjects. The final numbers of subjects included, demographics and descriptive statistics of the parameters took into consideration are shown in Table 1.

**Table 1:** Daemographics. Basic Measurements. Comorbidities.

	Controls	HFpEF	HFmrEF	HFrEF	P Value
Patients in group, n	17	18	21	20	
Age, y	63.9 ± 6.9	71.5 ± 7.1	68.3 ± 9.4	64.7 ± 9.2	0.12
Male subjects	9	9	13	12	0.75
Heart rate (bpm)	60.2 ± 9.4	63.5 ± 9.1	65.8 ± 8.5	66.1 ± 10.0	0.23
LVEDV (ml)	126 ± 31	120 ± 31	173 ± 32	244 ± 73	<0.001^§^
LVEDVi (ml/m^2^)	64 ± 11	62 ± 14	90 ± 15	116 ± 31	<0.001^§^
LVESV (ml)	45 ± 16	48 ± 17	96 ± 20	164 ± 51	<0.001^§^
LVESVi (ml/m^2^)	25 ± 2	27 ± 9	53 ± 11	91 ± 28	<0.001^§^
LV Stroke Volume (ml)	81 ± 16	72 ± 18	77 ± 14	80 ± 26	0.87
LA Surface (cm^2^)	20.4 ± 4.1	21.1 ± 6.0	23.1 ± 6.8	27.7 ± 6.1	0.003^§^
RA Surface (cm^2^)	22.4 ± 5.2	21.6 ± 6.0	20.1 ± 4.5	21.6 ± 4.8	0.65
LVEF (%)	64.4 ± 5.2	60.0 ± 7.2	44.2 ± 3.0	32.3 ± 6.0	<0.001^§^
LV-EDD(mm)	49.1 ± 4.4	50.7 ± 4.3	57.4 ± 5.9	65.4 ± 7.5	<0.001^§^
Septum(mm)	9.2 ± 1.7	11.2 ± 2.2	11.7 ± 1.7	11.8 ± 3.0	<0.001^§^
Lateral Wall(mm)	7.2 ± 1.0	7.4 ± 1.5	7.1 ± 1.7	8.1 ± 2.2	0.35
LVM (g)	93 ± 24	83 ± 23	112 ± 38	143 ± 40	<0.001^§^
LVMi (g/m^2^)	47.4 ± 9.2	43.4 ± 8.9	104.0 ± 48.3	131.2 ± 50.5	<0.001^§^
E/e′ ratio	10.9 ± 3.9	11.1 ± 4.3	11.1 ± 1.6	14.8 ± 6.2	0.09
NT-proBNP (ng/l)	94 ± 62	451 ± 512	885 ± 1146	2198 ± 3479	0.007^§^
**Laboratory Values**					
Hemoglobin (g/dl)	13.9 ± 1.1	13.0 ± 1.3	13.7 ± 1.1	15.0 ± 1.1	
Hematocrit	0.40 ± 0.03	0.38 ± 0.03	0.40 ± 0.03	0.44 ± 0.04	
Creatinin (mg/dl)	0.87 ± 0.20	0.92 ± 0.18	1.06 ± 0.33	1.10 ± 0.39	
GFR (ml/min)	81 ± 10	71 ± 15	71 ± 18	71 ± 21	
Troponin T (ng/l)	7 ± 3	20 ± 17	19 ± 20	19 ± 12	
CRP (mg/dl)	1.3 ± 1.4	2.9 ± 2.5	2.9 ± 4.1	1.0 ± 0.7	
WBC (n/nl)	6.1 ± 1.6	7.1 ± 2.3	8.4 ± 2.3	8.5 ± 2.4	
**Comorbidities**, **risk factors**					
CAD	0	12	14	11	<0.001^§^
Peripheric Artery Disease	0	5	3	2	0.11
Hypertension	6	12	15	12	0.12
Diabetes	2	4	5	3	0.72
Hypercholesterolemia	4	12	14	11	0.031^§^
COPD	0	1	3	1	0.34
Smokers	6	7	8	6	0.94

LVEDV – left ventricle end diastolic volume, LVEDVi – left ventricle end diastolic volume index, LVESV – left ventricle end systolic volume, LVEF – LV ejection fraction, LV-EDD – left ventricle end diastole diameter, LA – left atrium, RA – right atrium, LVM – left ventricle mass, LVMi – left ventricle mass index, CAD – coronary artery disease, COPD – Chronic obstructive pulmonary disease. Values are given as mean ± SD unless stated otherwise. Comparison between groups are assessed through an ANOVA 1-way. P values were adjusted with the Bonferroni method, a level above 0.05 is considered significant. ^§^Significant.

### Cardiac magnetic resonance

All CMR images were acquired using a 1.5 T (Achieva, Philips Healthcare, Best, The Netherlands) MRI scanners with a 5-channel cardiac surface coil in a supine position. All study participants were scanned using identical comprehensive imaging protocol. The study protocol included initial scouts to determine cardiac imaging planes. Cine images were acquired using ECG-gated bSSFP sequence with multiple breath-holds at end-expiration in three left ventricular (LV) long-axis (two-chamber (2Ch), three-chamber (3Ch) and four-chamber (4Ch)) planes. The ventricular two-chamber and four-chamber planes were used to plan a stack of short-axis slices covering the entire LV. The following imaging parameters were used: for 1.5 T scanner: repetition time (TR) = 3.3 ms, echo time (TE) = 1.6 ms, flip angle = 60°, voxel size = 1.8 × 1.7 × 8.0 mm^3^ and 50 phases per cardiac cycle in accordance with standards of procedure established in our unit and described previously^15^.

### Image analysis

**Figure 1:**
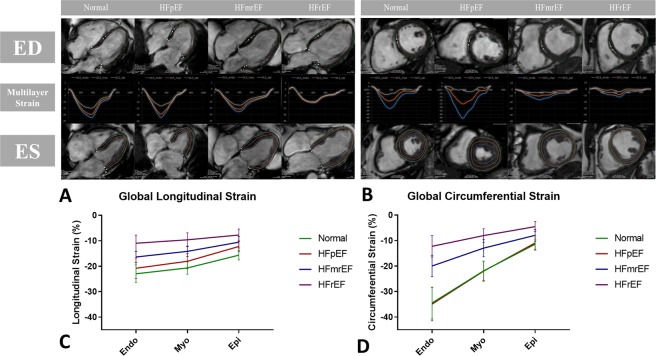
Range Variability of Multilayer Myocardial Strain across Different Stages of Heart Failure. (**A**) CMR Feature Tracking Assessment of Multilayer Longitudinal Strain: multilayer Longitudinal Strain in representative subjects from the 4 pathology groups, from left to right: Normal, HFpEF, HFmrEF, HFrEF. For each case on the vertical, from up to down, are shown respectively:long- axis 4Ch Cine Image in end-diastole, Global Longitudinal Strain vs time curves at 3 distinct myocardial layers, underlined Endo-, Myo- and Epi- myocardial layers at which the strain values were assessed, long-axis 4Ch Cine Image in end-systole. (**B**) CMR Feature Tracking Assessment of Multilayer Circumferential Strain: multilayer Circumferential Strain in the same representative subjects from the 4 groups, from left to right: Normal, HFpEF, HFmrEF, HFrEF. For each case on the vertical, from up to down, are shown, respectively: short-axis Basal Cine Image in end-diastole, Global Circumferential Strain vs time curves at 3 distinct myocardial layers, underlined Endo-, Myo- and Epi- myocardial layers at which the strain values were assessed, short-axis Basal Cine Image in end-systole. (**C**) Global Longitudinal Srain and (**D**) Global Circumferential Strain across the 4 pathology groups: multilayer assessment of Endo-, Myo-, Epi- layers of left ventricular myocardium. **Normal** () normal age-, gender- matched control, **HFpEF** () patients with HF with preserved EF, **HFmrEF** () patients with HF with mid-range reduced EF, **HFrEF** () patients with HF with reduced EF.

All images were analyzed offline using commercially available software (Medis Suite, version 3.1, Leiden, The Netherlands) in accordance to recent consensus document for quantification of LV function using CMR^16^. Strain analysis included 2Ch, 3Ch and 4Ch cine images, and respectively, 3 preselected slices from the LV short-axis stack to correspond to basal, mid-ventricular and apical levels. The endocardial and epicardial contours drawn on cine images with QMass version 8.1 were transferred to QStrain RE version 2.0, where, after the application of tissue tracking algorithm, endocardial and epicardial borders were detected throughout all the cardiac cycle. These long-axis cine images were further used to compute global myocardial longitudinal (GLS) (Fig. 1A) and, respectively, short-axis images were used to compute global circumferential (GCS) (Fig. 1B) strain and strain-rate at 3 distinct levels within the myocardial volume: Endo- subendocardial myocardium, Myo- mid-myocardium, and Epi- subepicardial myocardium. The global values for each layer were respectively obtained through averaging the values according to an American Heart Association (AHA) 17 segments model^17^, apex being excluded, as follows: GCS from averaging CS for 6 basal, 6 mid and 4 apical segmental individual values; GLS from 2Ch, 3Ch and 4Ch averaging 6 basal, 6 mid and 4 apical segments using a bull-eye view algorithm. For ROC analyses, individual values of peak strain corresponding to each 17 AHA segment, apex excluded, was rendered by the Medis platform and these values were individually considered and included in the statistical analysis. We assessed also the global values of radial strain in all subjects included in the study (data presented in the Supplemental Material), however our findings, in agreement with previous studies^18^, show a lower reproducibility and less sensitivity to detect differences in myocardial deformation between groups, suggesting further its reduced potential role in clinical practice.

LV Volumetry and mass were derived from segmentation of endocardial and epicardail contours in end-diastole and end-systole phases respectively, following the current guidelines recommendations^19^. Atrial surfaces were derived from the CMR cine sequences corresponding to the maximal expansion of the atrial chambers following the current guidelines^20^. For quantitative analysis all the volumes, areas and mass were indexed with BSA.

### Statistical analysis

Statistical analysis was carried out with SPSS IBM statistics, version 25. Normality of variables was assessed by visual assessment of normality curves and the Shapiro-Wilk test. Intra-subject comparison between strain values at 3 different levels Endo-, Myo-, Epi- were performed using 1-way repeated measures ANOVA and respectively Tukey’s multiple comparison tests. Comparison between groups for continuous variables was performed with a 2-sided, independent-samples Student’s t test or 1-way ANOVA for normally distributed data and Mann-Whitney and Kruskal-Wallis tests for skewed data. When a significant p value was obtained using 1-way ANOVA, the group means were examined by unpaired t tests for normally distributed variables or log-transformed values (if non-normally distributed), followed by Bonferroni post-hoc correction. ROC analysis with determination of specific area under curve (AUC) and threshold Youden’s index to establish optimal predictive power were computed. Results are presented as mean ± SD. Values of p < 0.05 were considered statistically significant.

## Results

### Baseline characteristics

A total number of 140 patients with a diagnosis of HF were screened for the inclusion criteria, as stated above, and a resultant number of 59 patients were finally recruited for the study, as follows: 18 were diagnosed with HFpEF, 21 with HFmrEF and 20 with HFrEF. Patients with AF, haemodynamically significant valvulopathies, uncontrolled hypertension, generic contraindications for CMR such as implantable devices, severe renal insufficiency, claustrophobia, were excluded. There were no differences between the control group and HFpEF for LV end-diastolic or end-systolic volumes or ejection fraction, as expected LV end-diastolic volume was progressively larger in patients with HFmrEF and HFrEF respectively. Patients with HFmrEF and HFrEF had a more dilated ventricle and left atrium. LV wall thickness was slightly larger in all 3 HF groups compared with normal measured at septum level there was no difference in the free wall, LV mass was higher in HFmrEF and HFrEF groups but there was no difference between HFpEF and Normal. Incidence of relevant comorbidities demonstrated a uniform distribution between the pathology groups (details are shown in Table 1).

### Longitudinal strain. Comparison between the the 4 groups

**Figure 2:**
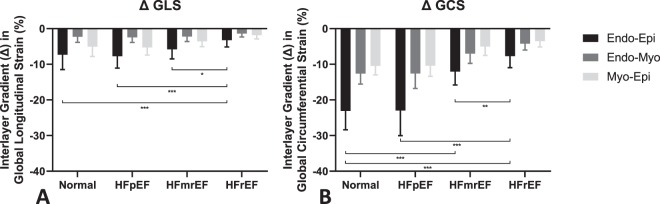
Interlayer Strain Gradient. Representation of (**A**) Global Longitudinal Strain and (**B**) Global Circumferential Strain Interlayer gradient (Δ) between Endo-Epi, Endo-Myo and Myo-Epi respectively in 4 groups: Normal – normal age-, gender- matched control, HFpEF – patients with HF with preserved EF, HFmrEF – patients with HF with mid-range reduced EF, HFrEF – patients with HF with reduced EF.

We initially looked for intrasubject paired comparisons between Endo-, Myo- and Epi- layers of the myocardium. Both GLS and GLSR, decreased progressively with an Endo-Myo-Epi gradient, p < 0.001. (means and standard errors for the 4 pathology groups are presented in Table 2). We further looked at the gradient between interlayer gradient across the groups: Endo-Epi gradient decreased between Normal and HFrEF (ΔGLS = −7.3 ± 4.2 vs −3.2 ± 1.9, p < 0.001) and respectively between HFmrEF and HFrEF (ΔGLS = −5.8 ± 2.7 vs −3.2 ± 1.9, p = 0.045), but was not different between Normal and HFpEF (ΔGLS = −7.3 ± 4.2 vs −7.8 ± 3.3) (Fig. 2A).

**Table 2:** Global Logitudinal and Circumferential Strain and Strain-Rate.

	Controls	HFpEF	HFmrEF	HFrEF	^#^P Value Controls vs HFpEF	^#^P Value Controls vs HFmrEF	^#^P Value HFpEF vs HFmrEF	^#^P Value HFmrEF vs HFrEF
**Global Longitudinal Strain**								
Endo- (%)	−23.0 ± 3.5	−20.0 ± 3.3	−16.4 ± 2.2	−11.0 ± 3.2	0.020	<0.001	<0.001	<0.001
Myo- (%)	−20.7 ± 2.4	−17.5.0 ± 2.6	−14.5 ± 2.1	−9.6 ± 2.7	0.001	<0.001	<0.001	<0.001
Epi- (%)	−15.7 ± 1.9	−12.2 ± 2.1	−10.6 ± 2.3	−7.7 ± 2.3	<0.001	<0.001	0.044	<0.001
^†^P Value Repeated Measure ANOVA in Pathology Groups	<0.001	<0.001	<0.001	<0.001				
**Global Circumferential Strain**								
Endo- (%)	−34.5 ± 6.2	−33.9 ± 5.7	−20.0 ± 4.2	−12.3 ± 4.2	0.51	<0.001	<0.001	<0.001
Myo- (%)	−21.9 ± 3.8	−21.3 ± 2.2	−13.0 ± 3.4	−8.0 ± 2.7	0.39	<0.001	<0.001	<0.001
Epi- (%)	−11.4 ± 2.0	−10.9 ± 2.3	−7.9 ± 2.3	−4.5 ± 1.9	0.54	<0.001	<0.001	<<0.001
**Global Longitudinal** **Strain Rate**								
Endo- (%/s)	−1.18 ± 0.19	−1.13 ± 0.30	−0.85 ± 0.16	−0.58 ± 0.13	0.52	<0.001	<0.001	<0.001
Myo- (%/s)	−1.05 ± 0.14	−0.94 ± 0.20	−0.75 ± 0.13	−0.50 ± 0.11	0.089	<0.001	<0.001	<0.001
Epi- (%/s)	−0.84 ± 0.11	−0.71 ± 0.15	−0.60 ± 0.10	−0.46 ± 0.17	0.008	<0.001	0.010	<0.001
^†^P Value Repeated Measure ANOVA in Pathology Groups	<0.001	<0.001	<0.001	<0.001				
**Global Circumferential** **Strain Rate**								
Endo- (%/s)	−1.79 ± 0.41	−1.86 ± 0.54	−1.02 ± 0.27	−0.72 ± 0.20	0.68	<0.001	<0.001	<0.001
Myo- (%/s)	−1.14 ± 0.20	−1.12 ± 0.19	−0.70 ± 0.16	−0.53 ± 0.16	0.76	<0.001	<0.001	0.002
Epi- (%/s)	−0.70 ± 0.13	−0.69 ± 0.08	−0.51 ± 0.13	−0.40 ± 0.17	0.74	<0.001	<0.001	0.005
^†^P Value Repeated Measure ANOVA in Pathology Groups	<0.001	<0.001	<0.001	<0.001				

Endo- sub-endocardial myocardium layer, Myo- mid-myocardial layer, Epi- sub-epicardial myocardium layer, Controls – normal control volunteers, HFpEF – heart failure with preserved ejection fraction, HFmrEF – heart failure with mid-range ejection fraction, HFrEF – heart failure with reduced ejection fraction. Comparison between pathology groups are assessed through an ANOVA 1-way. ^#^P values characterize multiple mean comparisons between groups, adjusted with the Bonferroni method, ^†^P values characterize repeated measures ANOVA for intrasubject comparisons. A P value level above 0.05 is considered significant.

We further compared the GLS values between the groups at every of the 3 multilayer levels between the 4 pathology groups. (results are summarized in Tabel 2). GLS was able to discriminate between Normal and HFpEF groups (Endo-: −23.0 ± 3.5 vs −20.0 ± 3.3, p = 0.020; Myo-: −20.7 ± 2.4 vs −17.5.0 ± 2.6, p = 0.001; Epi-: −15.7 ± 1.9 vs −12.2 ± 2.1, p < 0.001) (Fig. 1C). In contrast, GLSR was not different at Endo- level, showed a trend of decrease at Myo- level and is significant at Epi- level (−0.84 ± 0.11 vs −0.71 ± 0.15, p = 0.008) between these 2 groups. GLS and GLSR at all three levels was significantly higher in Normal than in patients with HFmrEF and HFrEF and, respectively, higher in HFmrEF than in the HFrEF, indicating a progressive loss of long axis contraction force in systolic HF.

### Circumferential strain. Comparison between the 4 groups

Similarly GCS and GCSR intrasubject paired comparisons between Endo-, Myo- and Epi- layers of the myocardium showed a progressive decrease (p < 0.001, means and standard errors for the 4 pathology groups are presented in Table 2). Endo-Epi gradient decreased significantly in patients with systolic HF from Normal to HFmrEF and, progressively more in HFrEF (ΔGCS = −23.1 ± 5.3 vs −12.1 ± 3.8 vs −7.7 ± 3.2, p < 0.001 between Normal and HFmrEF and between HFmrEF and HFrEF respectively, p = 0.033) (Fig. 2B). GCS and GCSR showed no difference in any layer between Normal and HFpEF, but in contrast, GCS and GCSR decreased significantly between Normal and HFmrEF and between HFmrEF and HFrEF groups (Fig. 1D).

### Segmental analysis of GLS and GCS. Regional variability of strain values

**Figure 3:**
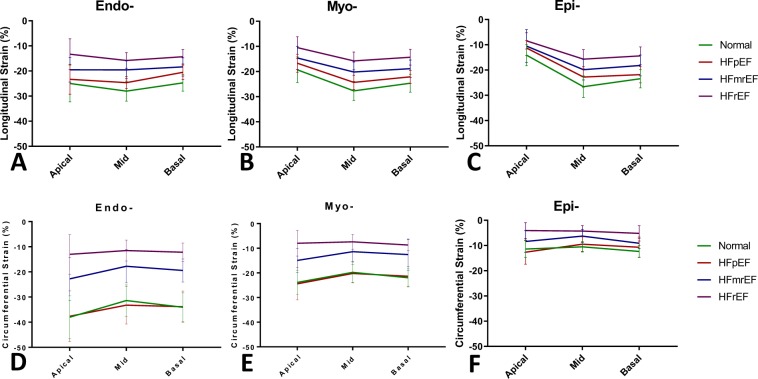
Regional Values of GLS and GCS across the 3 groups of patients with HF and Normal. (**A**) Endo-, (**B**) Myo- and (**C**) Epi- multilayer assessment of regional longitudinal strain and respectively (**D**) Endo-, (**F**) Myo- and (**E**) Epi- multilayer assessment of regional circumferential strain of left ventricular myocardium at 3 distinct ventricular levels Apical, Mid-ventricular and Basal in 4 groups: Normal – normal age-, gender- matched control, HFpEF – patients with HF with preserved EF, HFmrEF – patients with HF with mid-range EF, HFrEF – patients with HF with reduced EF.

To establish if there are significant differences in the GLS and GCS respectively between the base, mid-ventricular and apical segments, we used the segmental strain values for the 17 AHA model segments, apex excluded, per each individual subject to compare the cumulated values for the 6 basal, 6 mid-ventricular and 4 apical segments. A total number of 1292 segments were included in our analysis. To obtain strain values that correspond to a sub-region of the ventricle the strain values of corresponding segments were averaged accordingly. Results are summarized in Table 3 and represented in Fig. 3). In the Endo- layer, GLS had similar values between apex, mid-ventricle and base but in the Myo- and Epi- layers was lower in the apical segments in all 4 groups (P < 0.001) (Fig. 3A–C). GCS varied in amplitude with highest values apically and lowest at mid-ventricular level in normal subjects in Endo- and Myo- layers (Endo-: apical: −38.0 ± 8.5, mid-ventricle: −31.4 ± 6.3, basal: −34.1 ± 5.7, Myo-: apical: −23.8 ± 4.8, mid-ventricle: −19.8 ± 4.1, basal: −22.0 ± 3.7, p < 0.001) but not in Epi- layer. A similar distribution pattern was present in the other groups, with the lowest mean in the mid-ventricular segments but the difference between these groups did not reach significance (Fig. 3D–F).

**Table 3:** Regional Longitudinal and Circumferential Strain.

	Controls	HFpEF	HFmrEF	HFrEF	P Value Controls vs HFpEF	P Value Controls vs HFmrEF	P Value HFpEF vs HFmrEF	P Value HFmrEF vs HFrEF
**Regional Longitudinal Strain - Basal Segments**								
Endo- (%)	−24.7 ± 3.3	−20.5 ± 2.9	−18.4 ± 3.7	−14.3 ± 2.9	<0.001	<0.001	0.06	0.005
Myo- (%)	−24.7 ± 3.5	−22.1 ± 2.6	−18.9 ± 3.5	−14.3 ± 3.1	0.029	<0.001	0.005	<0.001
Epi- (%)	−23.4 ± 3.6	−21.8 ± 3.2	−18.1 ± 4.1	−14.4 ± 3.5	0.15	<0.001	0.01	0.004
**Regional Longitudinal Strain - Mid Ventricle Segments**								
Endo- (%)	−28.0 ± 4.0	−24.7 ± 2.4	−19.6 ± 3.6	−15.8 ± 3.1	0.006	<0.001	<0.001	0.001
Myo- (%)	−27.7 ± 3.8	−24.3 ± 2.8	−20.2 ± 4.0	−15.8 ± 3.5	0.008	<0.001	<0.001	<0.001
Epi- (%)	−26.6 ± 4.3	−22.8 ± 4.0	−19.9 ± 4.1	−15.7 ± 3.8	0.019	<0.001	0.024	0.003
**Regional Longitudinal Strain - Apical Segments**								
Endo- (%)	−25.0 ± 7.3	−23.3 ± 5.7	−19.5 ± 4.9	−13.3 ± 6.1	0.81	0.031	0.016	0.002
Myo- (%)	−19.3 ± 5.1	−16.7 ± 3.6	−14.6 ± 4.6	−10.5 ± 4.3	0.17	0.009	0.18	0.006
Epi- (%)	−14.1 ± 4.2	−11.2 ± 3.0	−10.5 ± 6.5	−8.4 ± 3.2	0.018	0.064	0.82	0.014
**Regional Circumferential Strain - Basal Segments**								
Endo- (%)	−35.3 ± 6.0	−35.3 ± 5.6	−22.1 ± 4.2	−14.7 ± 3.6	0.85	<0.001	<0.001	<0.001
Myo- (%)	−23.6 ± 3.7	−23.0 ± 3.8	−15.9 ± 2.7	−10.1 ± 2.2	0.54	<0.001	<0.001	<0.001
Epi- (%)	−15.6 ± 2.3	−14.2 ± 2.3	−12.1 ± 2.0	−9.2 ± 1.5	0.16	<0.001	0.006	<0.001
**Regional Circumferential Strain - Mid Ventricle Segments**								
Endo- (%)	−32.6 ± 6.0	−32.9 ± 6.8	−21.3 ± 4.8	−14.4 ± 4.1	0.88	<0.001	<0.001	<0.001
Myo- (%)	−21.3 ± 3.9	−21.3 ± 3.2	−14.6 ± 2.7	−10.4 ± 3.3	0.73	<0.001	<0.001	<0.001
Epi- (%)	−14.1 ± 2.2	−14.6 ± 3.9	−10.6 ± 2.1	−8.4 ± 2.7	0.73	<0.001	<0.001	0.003
**Regional Circumferential Strain - Apical Segments**								
Endo- (%)	−38.7 ± 8.3	−35.5 ± 9.1	−24.6 ± 7.7	−16.4 ± 7.3	0.32	<0.001	<0.001	0.001
Myo- (%)	−24.8 ± 4.4	−23.6 ± 4.4	−17.2 ± 4.9	−11.0 ± 5.1	0.44	<0.001	<0.001	<0.001
Epi- (%)	−14.6 ± 3.2	−16.1 ± 4.6	−12.6 ± 4.2	−8.3 ± 4.0	0.24	0.052	0.015	<0.001

Endo- sub-endocardial myocardium layer, Myo- mid-myocardial layer, Epi- sub-epicardial myocardium layer, Controls – normal control volunteers, HFpEF – heart failure with preserved ejection fraction, HFmrEF – heart failure with mid-range ejection fraction, HFrEF – heart failure with reduced ejection fraction. Comparison between pathology groups are assessed through an ANOVA 1-way. A P value level above 0.05 is considered significant.

### Segmental analysis of GLS and GCS. ROC analyses

**Figure 4:**
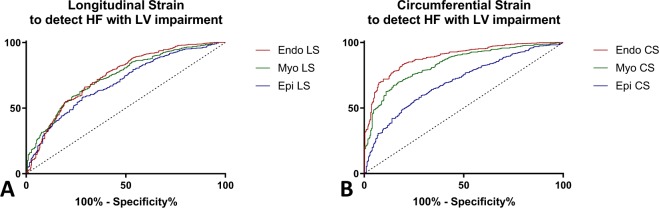
Comparative ROC Analysis for Multilayer GLS and GCS Parameters. ROC analysis to discriminate patients with HF with LV impairment from Normal Subjects of multilayer. (**A**) Longitudinal Strain, and (**B**) Circumferential Strain (Endo LS – sub-endocardial longitudinal strain, Myo LS – mid-myocardial longitudinal strain, Epi LS, sub-epicardial longitudinal strain, Endo CS – sub-endocardial circumferential strain, Myo CS – mid-myocardial circumferential strain, Epi CS, sub-epicardial circumferential strain).

In order to establish the sensitivity and specificity of each parameter analyzed to detect contractile impairment associated with HF, we derived the multilayer values of GLS and GCS for each individual segment, We then pooled the HFmrEF and HFrEF segments as patients with systolic heart failure and overt systolic impairment and compared this larger pool with the segments from normal controls. Quantified at a segmental level Endo- GCS showed the best combined specificity and sensitivity to discriminate contractile impairment with an AUC of 0.89, p < 0.001 (Fig. 4), while the for the Myo- GCS, AUC = 0.83 and for the Epi- GCS AUC = 0.70. At a segmental level, GLS has a similar discriminating capacity (Endo-GLS AUC = 0.74, Myo-GLS AUC = 0.74, Epi-GLS AUC = 0.69) (Table 4). A ROC curve analysis to identify patients with HFpEF from Normal, GLS Endo-, Myo- and Epi- showed low but statistically significant discriminating capacity. (data presented in Table 5 and Supplemental Table and Figures).

**Table 4:** ROC Analysis. Segmental and Global Longitudinal and Circumferential Strain to Predict Contractility Impairment in Patients with Systolic Heart Failure.

	AUC	Threshold	P Value for ROC Curve	Sensitivity, %	Specificity, %
**Segmental Longitudinal Strain**					
Endo-, %	0.74 (0.71–0.78)	−20.2	<0.0001	66	71
Myo-, %	0.74 (0.70–0.77)	−19.2	<0.0001	68	68
Epi-, %	0.69 (0.66–0.73)	−15.6	<0.0001	58	72
**Segmental Circumferential Strain**					
Endo-, %	0.89 (0.87–0.91)	−28.1	<0.0001	83	81
Myo-, %	0.83 (0.81–0.86)	−16.5	<0.0001	70	83
Epi-, %	0.70 (0.67–0.74)	−10.5	<0.0001	60	71
**Global Longitudinal Strain**					
Endo-, %	0.98 (0.94–1)	−19.4	<0.0001	98	88
Myo-, %	0.99 (0.99–1)	−16.4	<0.0001	95	100
Epi-, %	0.99 (0.96–1)	−13.0	<0.0001	95	100
**Global Circumferential Strain**					
Endo-, %	0.99 (0.96–1)	−27.1	<0.0001	100	88.2
Myo-, %	0.97 (0.94–1)	−17.7	<0.0001	98	88
Epi-, %	0.93 (0.87–1)	−9.3	<0.0001	90	88
**Other Parameters**					
LVM index (g/m^2^)	0.91 (0.82–0.99)	62.0	<0.0001	89	95
LVEDV index (ml/m^2^)	0.88 (0.78–0.97)	91.8	<0.0001	78	100
LA Area Index (cm^2^/m^2^)	0.68 (0.54–0.83)	24.5	0.032	46	94
RA Area Index (cm^2^/m^2^)	0.57 (0.40–0.74)		0.392		

Endo- sub-endocardial myocardium layer, Myo- mid-myocardial layer, Epi- sub-epicardial myocardium layer, AUC –area under curve, a P value level above 0.05 is considered significant, LVM – left ventricular mass, LVEDV – left ventricular end diastolic volume, LA- left atrium, RA – right atrium.

**Table 5:** Segmental and Global Longitudinal Strain to Predict Contractility Impairment in Patients with HFpEF.

	AUC	Threshold	P Value for ROC Curve	Sensitivity, %	Specificity, %
**Segmental Longitudinal Strain**					
Endo-, %	0.58 (0.54–0.63)	−27.1	0.0005	67	47
Myo-, %	0.58 (0.53–0.62)	−31.81	0.0013	89	24
Epi-, %	0.57 (0.52–0.62)	−15.6	0.0037	41	72
**Global Longitudinal Strain**					
Endo-, %	0.67 (0.49–0.84)	−24.1	0.0824	90	41
Myo-, %	0.78 (0.63–0.93)	−20.3	0.0038	90	59
Epi-, %	0.89 (0.78–0.99)	−13.0	<0.0001	65	100
**Other Parameters**					
LVM index (g/m^2^)	0.65 (0.46–0.85)		0.1502		
LVEDV index (ml/m^2^)	0.59 (0.38–0.80)		0.3880		
LA Area Index (cm^2^/m^2^)	0.72 (0.53–0.92)	21.50	0.0314	80	71
RA Area Index (cm^2^/m^2^)	0.54 (0.33–0.75)		0.7057		
E/e′	0.62(0.37–0.87)		0.360		

Endo- sub-endocardial myocardium layer, Myo- mid-myocardial layer, Epi- sub-epicardial myocardium layer, AUC –area under curve, a P value level above 0.05 is considered significant, LVM – left ventricular mass, LVEDV – left ventricular end diastolic volume, LA- left atrium, RA – right atrium.

### Scatterplot. Complementary role of GCS and GLS

**Figure 5:**
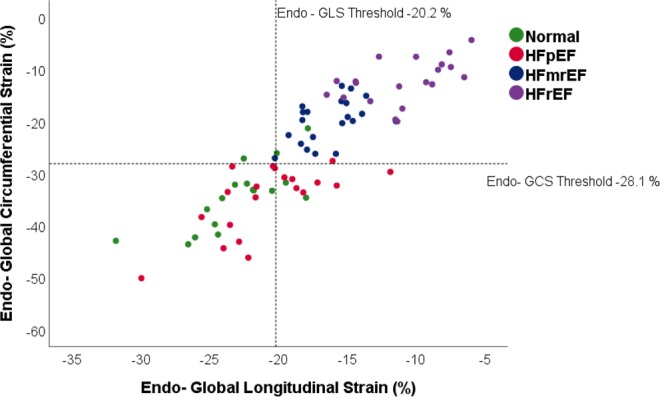
Distribution of HF Patients and Normal according to GLS and GCS Threshold Values. Scatterplot with Endo- Global Longitudinal Strain and Endo- Global Circumferential values. Reference lines represent the threshold values obtained through ROC analysis and Youden’s index calculation. The 4 groups represented are Normal – normal age-, gender- matched control, HFpEF – patients with HF with preserved EF, HFmrEF – patients with HF with mid-range reduced EF, HFrEF – patients with HF with reduced EF.

We further applied the threshold values of Endo- GLS and Endo GCS- resulted from the segmental ROC analysis to divide our cohort (Fig. 5) in 4 quadrants: subjects with Normal GCS and GLS values, subjects with abnormal GCS but normal GLS, subjects with abnormal GLS but normal GCS, subjects with abnormal GCS and abnormal GLS. The graph gives an indication of the excellent sensitivity of both GCS and GLS values to discriminate between normal and patients with systolic impairment, practically all the patients with HFmrEF and HFrEF having their GCS under the threshold values and all but one HFmrEF patient with lower GLS than the respective GLS threshold. Interestingly, the same Endo- GLS threshold applied to this specific subgroup, divides the HFpEF into 2 subgroups, suggesting that roughly a half of these patients have a degree of GLS impairment.

## Discussion

In this study, we applied CMR-FT to assess myocardial deformation at 3 distinct layers of the myocardium in patients with HF.

Our study findings can be summarized as follows:

- There is a marked heterogeneity of strain and strain values measured at the levels of sub-endocardial, mid and subepicardial myocardium with a significant positive gradient between endocardium (highest values) and epicardium (lowest values).
- This gradient is blunted in patients with more severely impaired EF suggesting the primary and proportionally more important loss of physiological contractile properties of the subendocardial myocardium.
- GCS is not different between HFpEF patients and normal controls in any of the myocardial layers and our data suggests that this parameter cannot be used to detect contractile impairment in these patients.
- GLS is on average significantly lower in HFpEF compared with Normal but this parameter is not specific and, in our cohort, almost half of the HFpEF patients had normal GLS. Corollary, our study proposes that a GLS threshold could be used to identifiy in the HFpEF group a subgroup of patients with putative significant underlying systolic impairment.
- Endo- GCS shows the best ability to diagnose patients with systolic HF and, in tandem with Endo- GLS, to identify with maximal sensitivity patients with systolic HF from our cohort.

Firstly, we report an important difference of magnitude for both GLS and GCS values depending of the level at which these parameters are assessed. In both cases, there is a significant endo-epi gradient with the highest values found towards the endocardium and the lowest towards the epicardium. Our findings support the hypothesis that subendocardial fibers play a privileged role in maintaining a physiological contractile power with a greater quantitative contribution, compared with fibers with myocardial and epicardial distribution. Proportionally, the strain values measured at an endocardial level dropped more with the severity of heart failure and myocardial remodeling. In keeping with this paradigm, we showed that measuring GCS at an endocardial level offers the best parameter to discriminate patients with systolic impairment, even if milder, such as HFmrEF group, from a normal population. These findings were in line with previous results of Altiok *et al*.^21^ who used comparatively speckle tracking and strain-encoded magnetic resonance imaging methodology to detect segmental hypokinesia.

Several echocardiography studies assessed myocardial strain using a multilayer approach in normal subjects^22–25^, coronary artery disease^26^, aortic stenosis^27^, hypertrophic cardiomyopathy^28,29^. There is a consensus among these studies regarding the existence of a marked gradient between subendocardial and subepicardial regions of the myocardium in normal subjects which decreases proportionally with the contractile impairment: in coronary artery disease (CAD) patients this gradient decreases with the severity of coronary obstruction^26^, in hypertension it is only mildly blunted compared with hypertrophic cardiomyopathy^28^.

The relative contribution of GCS and GLS to pump function of the heart, in normal and pathologic conditions, is controversial^30,31^. Histologically, circumferential fibers outnumber those with longitudinal and transversal orientation, especially at the base of the ventricle, with a ratio of 10:1^32^. Using a mathematical modeling of the LV, Stokke *et al*.^33^ argued that GCS is accountable for generating two times more contractile force than GLS and with a steeper positive correlation with the EF. Our data indicate higher absolute values of circumferential than longitudinal strain and a more severe decrease of GCS from normal towards patients with systolic heart failure (HFmrEF and HFrEF), *pari passu* with the decrease in EF.

Based on a segmental approach we propose generic threshold values for both GCS and GLS to identify patients with contractile impairment. We showed further excellent sensitivity of these combined values to identify patients with HF and, moreover, to scale the severity of disease, efficiently separating HFmrEF from HFrEF. While EF is a robust prognostic factor in a large number of patients with frank HF, with severely depressed cardiac contraction, its role per se in borderline subnormal and mid-range EF patients seems to be more controversial^34^. Recent ESC guidelines^14^ identifies HFmrEF as a potentially different pathologic entity from the more typical HFrEF group. HFmrEF class comprises patients with symptoms but not signs of HF, an intermediate degree of systolic dysfunction (EF between 40 and 50%) and with other traits of the disease present such as elevated natriuretic peptides, diastolic dysfunction, enlarged left atrium. Initial echocardiography studies in HFmrEF show mixed traits shared by both HFrEF and HFpEF such as the combination of LV dilatation with depressed contraction and respectively, increased stiffness and elevated diastolic filling pressures. Describing only discrete differences and similar patterns in strain values and interlayer gradients between HFmrEF and HFrEF, our study suggests that HFmrEF follows a similar pathophysiological pathway with HFrEF and likely represent a more incipient stage.

Overall, approximately one half of the total number of patients diagnosed with HF have preserved EF. Systolic contractility in patients with diastolic HF is insufficiently explored and understood^35^. Importantly, in our study, GCS in HFpEF group was not different from normal. In agreement with previous echocardiography studies^36,37^, we found that GLS is on average decreased in the HFpEF group compared with controls, however, due to a wider variation, its specificity to discriminate HFpEF patients from controls is low (Fig. 4). Our findings support the idea that an underlying systolic impairment in patients with primarily diastolic heart failure is inconstant and, where present, tends to affect only the long-axis systolic contraction.

### Clinical perspectives

The discrepancy in magnitude of strain values between endocardium and epicardium warrants a very careful approach to the resulting data. Despite representing a powerful tool to assess regional and global cardiac function, the strain imaging advent into day-by-day usage has been restricted so far by a modest concordance between methodologies to measure, compute and report these values.

Contractility is consistently impaired in patients with HFmrEF paralleling the degree of ventricular dilatation suggesting that drugs targeting the ventricular remodeling such as angiotensin converting enzyme inhibitors, Spironolactone or β-blockers maintain their beneficial effects in this group of patients still insufficiently characterized^38^.

### Limitations

We acknowledge several limitations of this study: (1) even if representative for a specific disease class, the groups of patients were small and more comprehensive studies are warranted to validate our findings in a widespread HF population, (2) in most of our patients the etiology of heart failure is mostly related to coronary artery disease and previous acute coronary events, an extrapolation of our findings to a more complex group of HF patients, especially those affected by an intrinsic cardiomyopathy, is warranted, (3) myocardial deformation can vary significantly between individual regions of the heart even in the absence of structural alterations^39^, therefore a segmental assessment of strain can be less sensitive in detecting patients with contractile impairment than global values.

## Conclusions

Multilayer evaluation of cardiac contraction is a convenient and fast procedure that can be obtained from basic CMR cine images. Measurement of strain and strain rate must be done cautiously as their range varies amply within the myocardial volume. Sub-endocardial regions and respectively circumferential strain seems to offer the best discriminative tool for the identification of patients with HF.

## Supplementary information

Supplementary Information

## References

[1] Merlo M (2014). Long-term prognostic impact of therapeutic strategies in patients with idiopathic dilated cardiomyopathy: changing mortality over the last 30 years. Eur J Heart Fail.

[2] Conrad N (2018). Temporal trends and patterns in heart failure incidence: a population-based study of 4 million individuals. Lancet.

[3] Writing Group M (2016). Heart Disease and Stroke Statistics-2016 Update: A Report From the American Heart Association. Circulation.

[4] Bogaert J, Rademakers FE (2001). Regional nonuniformity of normal adult human left ventricle. Am J Physiol Heart Circ Physiol.

[5] Buckberg G, Hoffman JI, Mahajan A, Saleh S, Coghlan C (2008). Cardiac mechanics revisited: the relationship of cardiac architecture to ventricular function. Circulation.

[6] Lorell BH, Carabello BA (2000). Left ventricular hypertrophy: pathogenesis, detection, and prognosis. Circulation.

[7] Urheim S, Edvardsen T, Torp H, Angelsen B, Smiseth OA (2000). Myocardial strain by Doppler echocardiography. Validation of a new method to quantify regional myocardial function. Circulation.

[8] Park JJ, Park JB, Park JH, Cho GY (2018). Global Longitudinal Strain to Predict Mortality in Patients With Acute Heart Failure. J Am Coll Cardiol.

[9] Stanton T, Leano R, Marwick TH (2009). Prediction of all-cause mortality from global longitudinal speckle strain: comparison with ejection fraction and wall motion scoring. Circ Cardiovasc Imaging.

[10] Eitel I (2018). Cardiac Magnetic Resonance Myocardial Feature Tracking for Optimized Prediction of Cardiovascular Events Following Myocardial Infarction. JACC Cardiovasc Imaging.

[11] Kempny A (2012). Quantification of biventricular myocardial function using cardiac magnetic resonance feature tracking, endocardial border delineation and echocardiographic speckle tracking in patients with repaired tetralogy of Fallot and healthy controls. J Cardiovasc Magn Reson.

[12] Kowallick JT (2016). Inter-study reproducibility of left ventricular torsion and torsion rate quantification using MR myocardial feature tracking. J Magn Reson Imaging.

[13] Morton G (2012). Inter-study reproducibility of cardiovascular magnetic resonance myocardial feature tracking. J Cardiovasc Magn Reson.

[14] Ponikowski P (2016). ESC Guidelines for the diagnosis and treatment of acute and chronic heart failure: The Task Force for the diagnosis and treatment of acute and chronic heart failure of the European Society of Cardiology (ESC)Developed with the special contribution of the Heart Failure Association (HFA) of the ESC. Eur Heart J.

[15] Lapinskas T (2018). Fatty metaplasia quantification and impact on regional myocardial function as assessed by advanced cardiac MR imaging. MAGMA.

[16] Suinesiaputra A (2015). Quantification of LV function and mass by cardiovascular magnetic resonance: multi-center variability and consensus contours. J Cardiovasc Magn Reson.

[17] Cerqueira MD (2002). Standardized myocardial segmentation and nomenclature for tomographic imaging of the heart. A statement for healthcare professionals from the Cardiac Imaging Committee of the Council on Clinical Cardiology of the American Heart Association. Circulation.

[18] Schmidt B (2017). Intra- and inter-observer reproducibility of global and regional magnetic resonance feature tracking derived strain parameters of the left and right ventricle. Eur J Radiol.

[19] Kawel-Boehm N (2015). Normal values for cardiovascular magnetic resonance in adults and children. J Cardiovasc Magn Reson.

[20] Maceira AM, Cosin-Sales J, Roughton M, Prasad SK, Pennell DJ (2010). Reference left atrial dimensions and volumes by steady state free precession cardiovascular magnetic resonance. J Cardiovasc Magn Reson.

[21] Altiok E (2012). Quantitative analysis of endocardial and epicardial left ventricular myocardial deformation-comparison of strain-encoded cardiac magnetic resonance imaging with two-dimensional speckle-tracking echocardiography. J Am Soc Echocardiogr.

[22] Adamu U, Schmitz F, Becker M, Kelm M, Hoffmann R (2009). Advanced speckle tracking echocardiography allowing a three-myocardial layer-specific analysis of deformation parameters. Eur J Echocardiogr.

[23] Alcidi GM (2018). Normal reference values of multilayer longitudinal strain according to age decades in a healthy population: A single-centre experience. Eur Heart J Cardiovasc Imaging.

[24] Leitman M (2010). Circumferential and longitudinal strain in 3 myocardial layers in normal subjects and in patients with regional left ventricular dysfunction. J Am Soc Echocardiogr.

[25] Shi J, Pan C, Kong D, Cheng L, Shu X (2016). Left Ventricular Longitudinal and Circumferential Layer-Specific Myocardial Strains and Their Determinants in Healthy Subjects. Echocardiography.

[26] Sarvari SI (2013). Layer-specific quantification of myocardial deformation by strain echocardiography may reveal significant CAD in patients with non-ST-segment elevation acute coronary syndrome. JACC Cardiovasc Imaging.

[27] Shiino Kenji, Yamada Akira, Scalia Gregory M., Putrino Anthony, Chamberlain Robert, Poon Karl, Walters Darren L., Chan Jonathan (2019). Early Changes of Myocardial Function After Transcatheter Aortic Valve Implantation Using Multilayer Strain Speckle Tracking Echocardiography. The American Journal of Cardiology.

[28] Sun JP (2019). Echocardiographic strain in hypertrophic cardiomyopathy and hypertensive left ventricular hypertrophy. Echocardiography.

[29] Vigneault DM (2019). Left Ventricular Strain Is Abnormal in Preclinical and Overt Hypertrophic Cardiomyopathy: Cardiac MR Feature Tracking. Radiology.

[30] Carlsson M (2018). Functional Contribution of Circumferential Versus Longitudinal Strain: Different Concepts Suggest Conflicting Results. J Am Coll Cardiol.

[31] Matthews SD, Rubin J, Cohen LP, Maurer MS (2018). Myocardial Contraction Fraction: A Volumetric Measure of Myocardial Shortening Analogous to Strain. J Am Coll Cardiol.

[32] Streeter DD, Spotnitz HM, Patel DP, Ross J, Sonnenblick EH (1969). Fiber orientation in the canine left ventricle during diastole and systole. Circ Res.

[33] Stokke TM (2017). Geometry as a Confounder When Assessing Ventricular Systolic Function: Comparison Between Ejection Fraction and Strain. J Am Coll Cardiol.

[34] Solomon SD (2005). Influence of ejection fraction on cardiovascular outcomes in a broad spectrum of heart failure patients. Circulation.

[35] Nagueh SF (2018). Classification of Left Ventricular Diastolic Dysfunction and Heart Failure Diagnosis and Prognosis. J Am Soc Echocardiogr.

[36] DeVore AD (2017). Impaired left ventricular global longitudinal strain in patients with heart failure with preserved ejection fraction: insights from the RELAX trial. Eur J Heart Fail.

[37] Kraigher-Krainer E (2014). Impaired systolic function by strain imaging in heart failure with preserved ejection fraction. J Am Coll Cardiol.

[38] Petutschnigg J, Edelmann F (2018). Heart failure with mid-range ejection fraction and with preserved ejection fraction. Herz.

[39] Tang X (2017). Left ventricular myocardial strain in ventricular arrhythmia without structural heart disease using cardiac magnetic resonance. Am J Transl Res.

