# Supplementary material for: Range Variability in CMR Feature Tracking Multilayer Strain across Different Stages of Heart Failure
Source: Sci Rep. 2019 Nov 11;9:16478. doi: 10.1038/s41598-019-52683-8 (PMC6848170; doi:10.1038/s41598-019-52683-8)
Supplement: Supplementary file 1 — Supplementary Information [file 41598_2019_52683_MOESM1_ESM.pdf]

# Supplementary Information

## Range Variability in CMR Feature Tracking Multilayer Strain across Different Stages of Heart Failure

Radu Tanacli [1], Djawid Hashemi [2,3], Tomas Lapinskas [1], Frank Edelmann [2,3], Rolf Gebker [1,3], Gianni Pedrizzetti [4], Andreas Schuster [5], Eike Nagel [6], Burkert Pieske [1,2,3], Hans-Dirk Düngen [2,3],

Sebastian Kelle [1,2,3]

[1] Department of Cardiology, German Heart Centre Berlin, Berlin, Germany

[2] Department of Cardiology, Charité University Medicine Berlin, Berlin, Germany.

[3] German Centre for Cardiovascular Research DZHK, Partner Site Berlin, Berlin, Germany.

[4]Department of Engineering and Architecture, University of Trieste, Trieste, Italy.

[5] Department of Cardiology and Pulmonology and German Centre for Cardiovascular Research (DZHK) Partner Site, Göttingen, Germany.

[6] Institute of Experimental and Translational Cardiac Imaging, DZHK Centre for Cardiovascular Imaging, Goethe University Hospital Frankfurt, Frankfurt am Main, Germany.

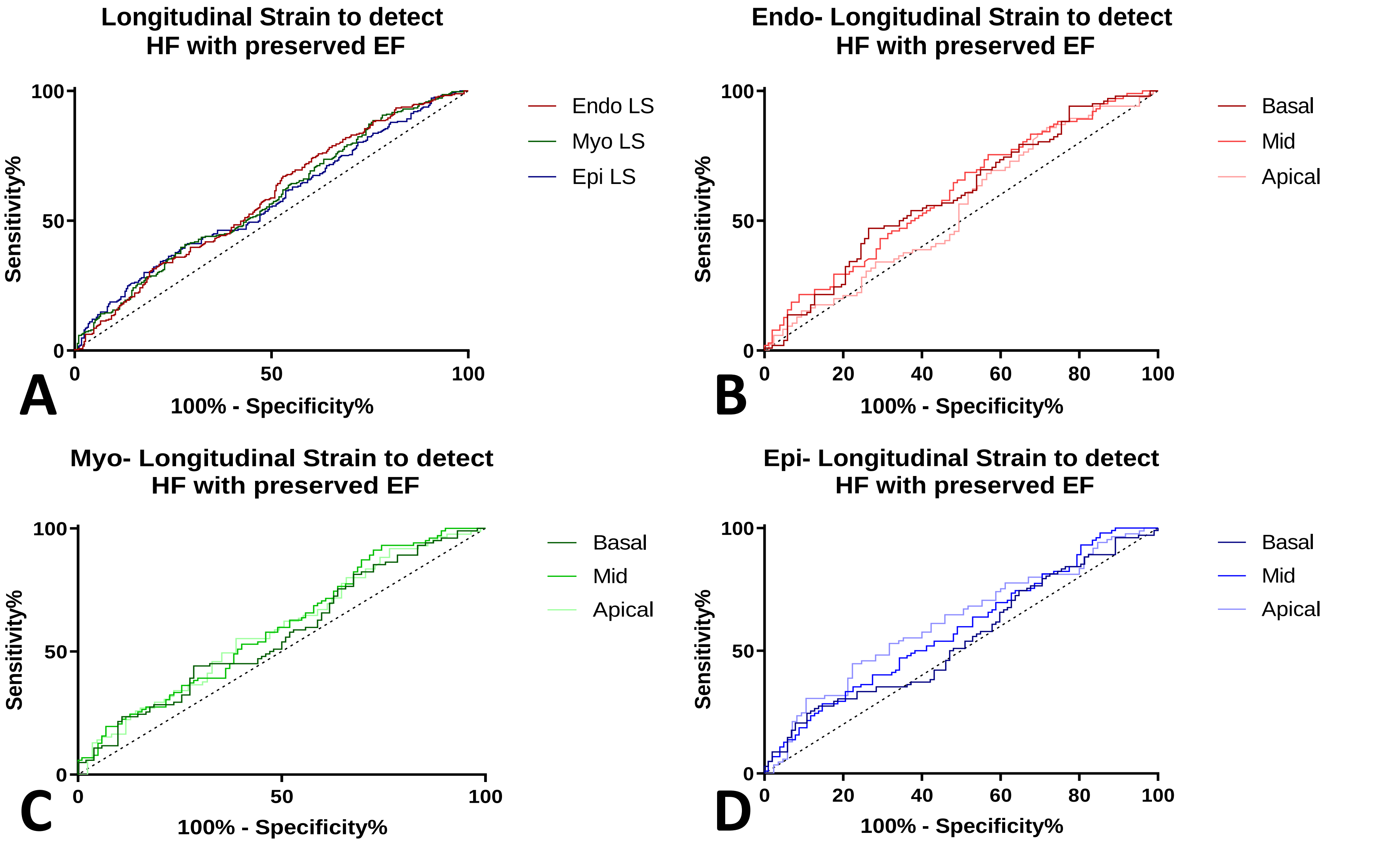


**Supplement Figure 1.** ROC analysis to discriminate patients with HF with preserved EF from Normal Subjects with multilayer longitudinal strain A) global segmental values, and B) regional basal segmental values,C) regional mid-ventricular segmental values, D)regional apical segmental values. (Endo LS – sub-endocardial longitudinal strain, Myo LS – mid-myocardial longitudinal strain, Epi LS, sub-epicardial longitudinal strain.

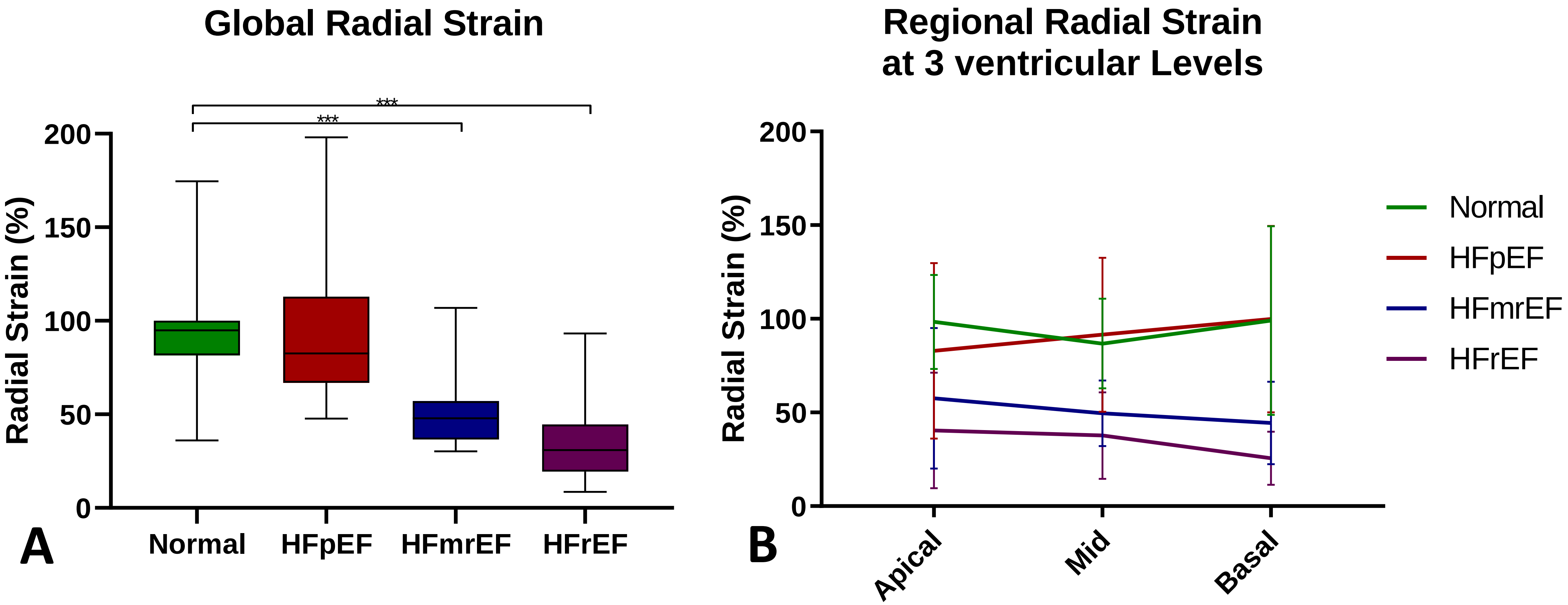


**Supplement Figure 2.** Monolayer assessment of A) Global and B) Regional Radial Strain. **Normal** – normal age-, gender- matched control, **HFpEF** – patients with HF with preserved EF, **HFmrEF** – patients with HF with mid-range reduced EF, **HFrEF** – patients with HF with reduced EF.

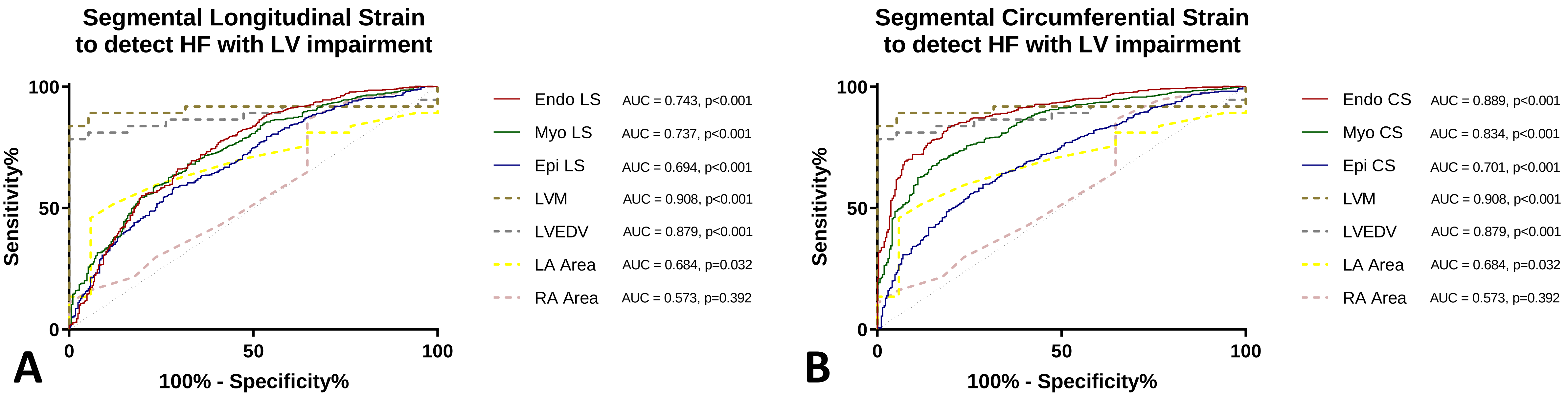


**Supplement Figure 3.** ROC analysis to discriminate patients with HF with LV impairment from Normal Subjects of multilayer A) Longitudinal Strain, and B) Circumferential Strain (Endo LS – sub-endocardial longitudinal strain, Myo LS – mid-myocardial longitudinal strain, Epi LS, sub-epicardial longitudinal strain, Endo CS – sub-endocardial circumferential strain, Myo CS – mid-myocardial circumferential strain, Epi CS, sub-epicardial circumferential strain ). Dotted lines represent respectively LVM – indexed left ventricular mass, LVEDV – indexed LV end-diastolic volume, LA and RA area – indexed left atrial and right atrial areas.

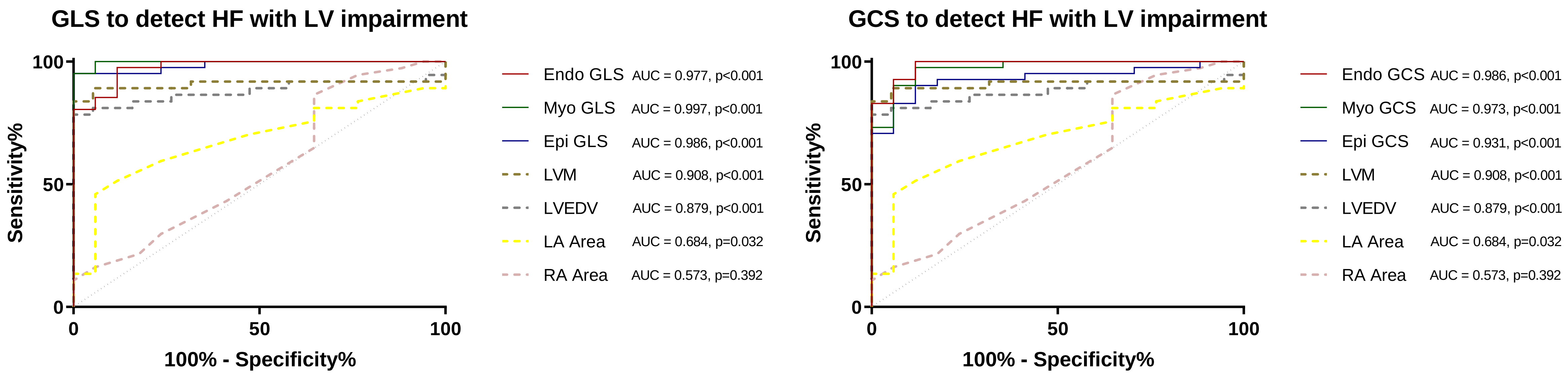


**Supplement Figure 4.** ROC analysis to discriminate patients with HF with LV impairment from Normal Subjects of multilayer A) GLS, and B) GCS(Endo GLS – sub-endocardial longitudinal strain, Myo GLS – mid-myocardial longitudinal strain, Epi GLS, sub-epicardial longitudinal strain, Endo GCS – sub-endocardial circumferential strain, Myo GCS – mid-myocardial circumferential strain, Epi GCS, sub-epicardial circumferential strain ). Dotted lines represent respectively LVM – indexed left ventricular mass, LVEDV – indexed LV end-diastolic volume, LA and RA area – indexed left atrial and right atrial areas.

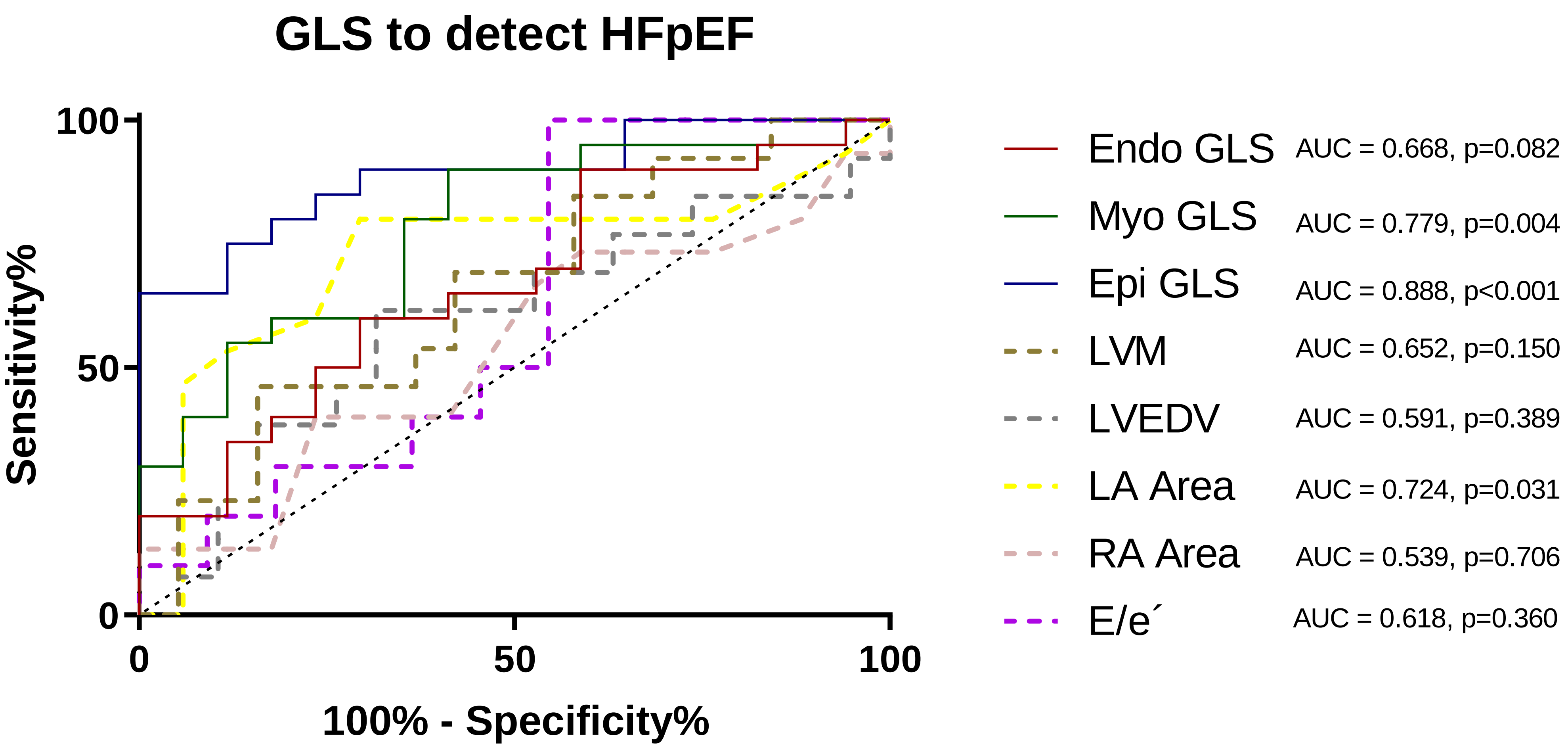


**Supplement Figure 5.** ROC analysis to discriminate patients with HF with preserved EF from Normal Subjects of multilayer A) Endo GLS – sub-endocardial longitudinal strain, Myo GLS – mid-myocardial longitudinal strain, Epi GLS, sub-epicardial longitudinal strain. Dotted lines represent respectively LVM – indexed left ventricular mass, LVEDV – indexed LV end-diastolic volume, LA and RA area – indexed left atrial and right atrial areas.

# Supplement Table. Global and Regional Radial Strain in the 4 pathology groups

| | Controls | HFpEF | HFmrEF | HFrEF | P# Value Controls vs HFpEF | P# Value Controls vs HFmrEF | P# Value HFpEF vs HFmrEF | P# Value HFmrEF vs HFrEF |
|---|---|---|---|---|---|---|---|---|
| Global Radial Strain (%) | | | | | | | | |
| | 94.8±28 | 91.4±36 | 50.5±19 | 34.6±20 | 0.98 | <0.001 | <0.001 | 0.22 |
| Global Radial Strain Rate (%/s) | | | | | | | | |
| | 2.68±0.1 | 2.71±0.2 | 1.75±0.2 | 1.39±0.3 | 0.99 | 0.0015 | 0.0012 | 0.17 |
| Regional Radial Strain (%) | | | | | | | | |
| Basal | 99.1±50 | 99.8±49 | 44.4±22 | 25.6±14 | 0.99 | <0.001 | <0.001 | 0.368 |
| Mid | 86.8±24 | 91.5±41 | 49.6±17 | 37.7±23 | 0.95 | <0.001 | <0.001 | 0.53 |
| Apical | 98.3±25 | 82.9±47 | 57.5±37 | 40.5±31 | 0.57 | 0.005 | 0.12 | 0.44 |
| Regional Radial Strain Rate (%/s) | | | | | | | | |
| Basal | 2.74±0.8 | 2.55±0.7 | 1.59±0.6 | 1.08±0.5 | 0.79 | <0.001 | <0.001 | 0.048 |
| Mid | 2.69±0.4 | 2.90±0.8 | 1.88±0.5 | 1.43±0.9 | 0.80 | 0.005 | <0.001 | 0.18 |
| Apical | 2.62±0.6 | 2.69±0.8 | 1.77±0.7 | 1.67±0.9 | 0.99 | 0.006 | 0.002 | 0.97 |

Normal – control subjects, HFpEF – patients with HF with preserved EF, HFmrEF patients with HF with mid-range reduced EF, HFrEF – patients with HF with reduced EF, Apical –apical segments, Mid – mid-ventricular segments, Basal – basal segments. A p value < 0.05 is considered significant.
